# Ellagitannin–Lipid Interaction by HR-MAS NMR Spectroscopy

**DOI:** 10.3390/molecules26020373

**Published:** 2021-01-12

**Authors:** Valtteri Virtanen, Susanna Räikkönen, Elina Puljula, Maarit Karonen

**Affiliations:** Natural Chemistry Research Group, Department of Chemistry, University of Turku, FI-20014 Turku, Finland; susanna.raikkonen@outlook.com (S.R.); elina.puljula@gmail.com (E.P.); maarit.karonen@utu.fi (M.K.)

**Keywords:** *E. coli*, HR-MAS-NMR, interaction, lipid membrane, tannins, UPLC-DAD-MS

## Abstract

Ellagitannins have antimicrobial activity, which might be related to their interactions with membrane lipids. We studied the interactions of 12 different ellagitannins and pentagalloylglucose with a lipid extract of *Escherichia coli* by high-resolution magic angle spinning NMR spectroscopy. The nuclear Overhauser effect was utilized to measure the cross relaxation rates between ellagitannin and lipid protons. The shifting of lipid signals in 1H NMR spectra of ellagitannin–lipid mixture due to ring current effect was also observed. The ellagitannins that showed interaction with lipids had clear structural similarities. All ellagitannins that had interactions with lipids had glucopyranose cores. In addition to the central polyol, the most important structural feature affecting the interaction seemed to be the structural flexibility of the ellagitannin. Even dimeric and trimeric ellagitannins could penetrate to the lipid bilayers if their structures were flexible with free galloyl and hexahydroxydiphenoyl groups.

## 1. Introduction

Tannins are a group of specialized plant metabolites, which, when included in the dietary feed of ruminants, have been shown to induce many beneficial effects such as increasing their effective amino acid absorption, lowering their methane production, and acting as anthelmintics [1,2,3,4,5,6]. Additionally, tannins have been recently shown to inhibit the growth of several bacteria more effectively than what they were previously thought capable [7]. Many of these favorable effects are traditionally thought to be governed by the protein affinity/protein precipitation capacity of tannins or their oxidative activity, which have been extensively studied in the literature [8,9,10]. However, the possible interactions between lipids and tannins have not been widely considered even though they might play an important role in understanding the capability and the mechanisms with which tannins inhibit, for instance, the growth of different bacteria and their possibilities as antimicrobial agents in general [11].

**Figure 1:**
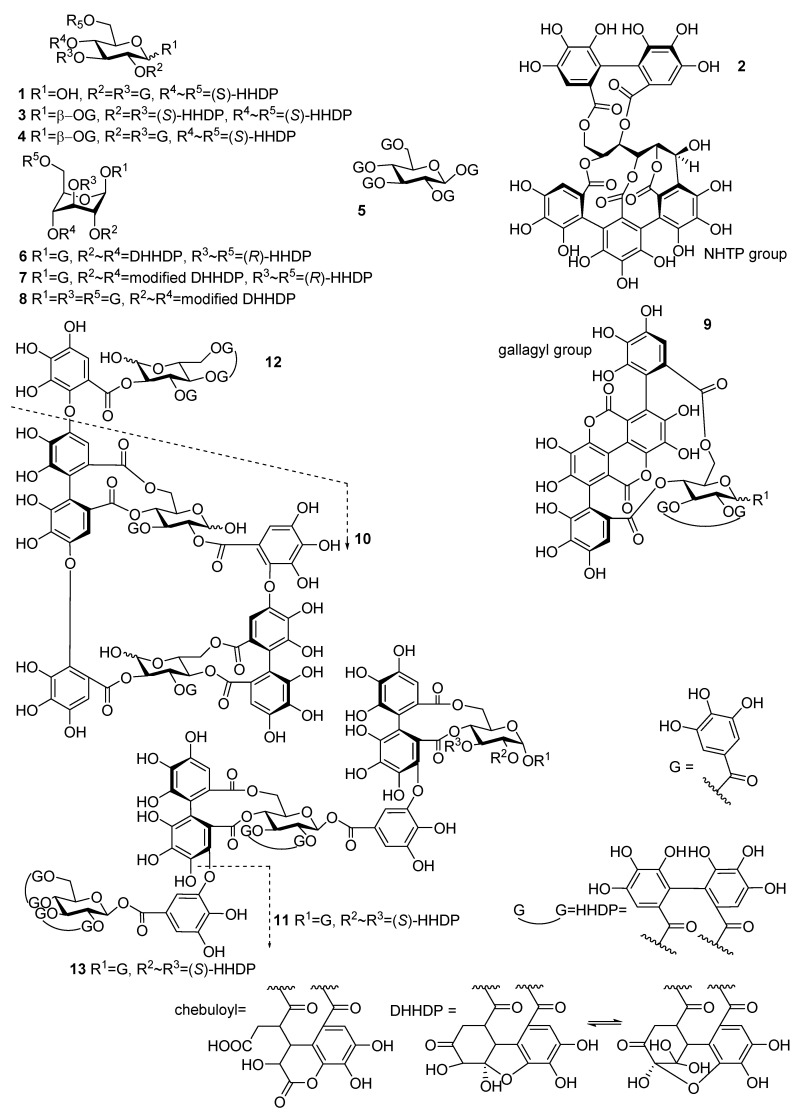
Chemical structures of 12 ellagitannins and pentagalloylglucose studied for their interaction with lipids: tellimagrandin I **1**, vescalagin **2**, casuarictin **3**, tellimagrandin II **4**, pentagalloylglucose **5**, geraniin **6**, chebulagic acid **7**, chebulinic acid **8**, punicalagin **9**, oenothein B **10**, sanguiin H-6 **11**, oenothein A **12**, and lambertianin C **13**. DHHDP = dehydrohexahydroxydiphenoyl, G = galloyl, HHDP = hexahydroxydiphenoyl, chebuloyl = modified dehydrohexahydroxydiphenoyl, NHTP = nonahydroxytriphenoyl.

High-resolution magic angle spinning (HR-MAS) NMR spectroscopy has revolutionized and opened all new possibilities to study lipids, lipid membranes, and their potential interactions with other compounds [12,13,14,15]. A notable benefit of the HR-MAS probe is that it tolerates the kind of semisolid emulsion type samples that ellagitannins (ETs) and lipids form in a solution, while still enabling normal liquid-state NMR experiments with reasonable resolution. Useful experiments include methods such as nuclear Overhauser effect spectroscopy (NOESY) to detect correlations between specific parts of the lipids with other molecules [12]. We studied the interactions of 12 ellagitannins (Figure 1) with a lipid extract of *Escherichia coli* (*E. coli*) by HR-MAS NMR. The ETs were selected to represent different branches of the ET biosynthetic pathway and based on their studied hydrophobicity [16,17,18]. In addition, the lipid interactions of pentagalloylglucose, the biosynthetic precursor of ETs, were studied. The main aims were to study whether there are interactions between ETs and lipids and whether these interactions can be studied by HR-MAS NMR.

## 2. Results and Discussion

### 2.1. Characterization of the Lipids in E. coli Lipid Extract

The protons in the *E. coli* lipid extracts were assigned mainly based on the 2D-correlation spectra measured with the 600 MHz instrument (Section 3.3), as the correlation spectra measured with the 400 MHz HR-MAS instrument did not achieve good enough resolution even after parameter optimization. Attempts were also made to analyze a sample of pure L-*α*-phosphatidylethanolamine (PE) lipid in D_2_O to verify the assignations made from the 400 MHz HR-MAS measurements, but, because of its poor solubility in water, these measurements did not produce the desired outcome. The solubility of the pure PE lipid was not markedly increased even after the addition of 0.1 M phosphate buffer or after mixing the pure PE lipid with different ratios of the *E. coli* lipid extract.

**Figure 2:**
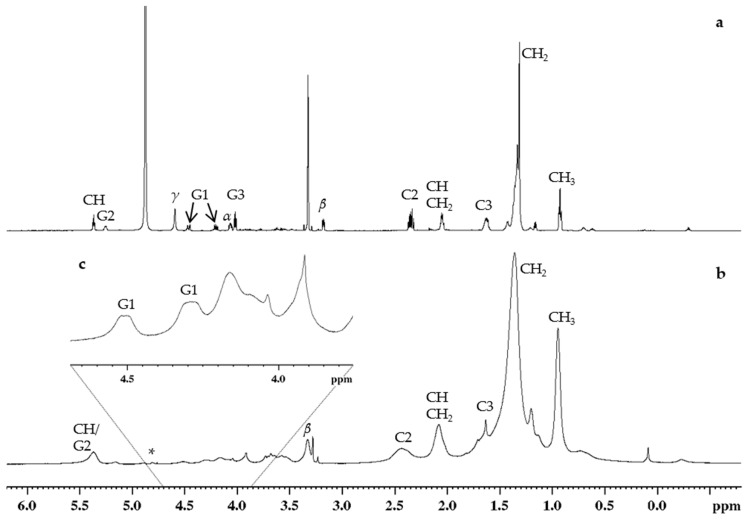
^1^H NMR spectra of the *E*. *coli* lipid extract with assigned proton signals at 25 °C: (**a**) measured in MeOD-*d*4 with 600 MHz, (**b**) measured in D_2_O with 400 MHz HR-MAS probe along with (**c**) a highlighted ppm range of 3.7–4.7. Example structures of the lipids with labels are presented in Figure 5. * Possible distortion in the HR-MAS spectra caused by water presaturation masks some signals detected in the 600 MHz spectra.

The assigned lipid protons are displayed in Figure 2 from spectra measured with both a 600 MHz instrument equipped with a Cryo-Probe (**a**) and a 400 MHz instrument equipped with an HR-MAS probe (**b**), and their chemical shifts are as follows: (**a**) ^1^H NMR (MeOD-*d*4, 600 MHz) δ 0.90 (m, H-CH_3_), 1.29 (m, H-CH_2_), 1.60 (m, H-C3), 2.03 (m, H-CHCH_2_), 2.33 (m, H-C2), 3.16 (m, H-*β*), 4.00 (m, H-G3), 4.05 (m, H-*α*), 4.19 (m, H-G1), 4.44 (m, H-G1), 4.57 (s, H-*γ*), 5.23 (m, H-G2), 5.35 (m, H-CH); and (**b**) ^1^H NMR (D_2_O, 400 MHz) δ 0.95 (m, H-CH_3_), 1.35 (m, H-CH_2_), 1.63 (m, H-C3), 2.08 (m, H-CHCH_2_), 2.44 (m, H-C2), 3.34 (m, H-*β*), 4.29 (m, H-G1), 4.51 (m, H-G1), 5.37 (m, H-G2/H-CH). Lipid protons H-G3, H-α, and H-γ could not be accurately assigned from the 400 MHz measurements owing to lower resolving power and the water suppression method used (described in Section 3.3), which masked the H-γ signal entirely. Additionally, the protons H-G2 and H-CH formed a single peak rather than resolving into two separate peaks in the 400 MHz HR-MAS spectrum.

### 2.2. ET–Lipid Interaction Measurements by HR-MAS NMR

**Figure 3:**
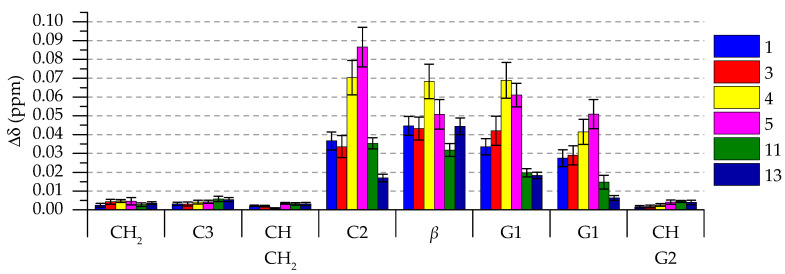
^1^H NMR chemical shift deltas (Δδ, ppm) of *E. coli* lipid extracts in the presence of tellimagrandin I **1**, casuarictin **3**, tellimagrandin II **4**, pentagalloylglucose **5**, sanguiin H-6 **11**, and lambertianin C **13**. The tannin numbering refers to Figure 1 and lipid proton assignations refer to Figure 5. Values are presented as Δδ (average values and standard error, n = 4).

**Figure A1:**
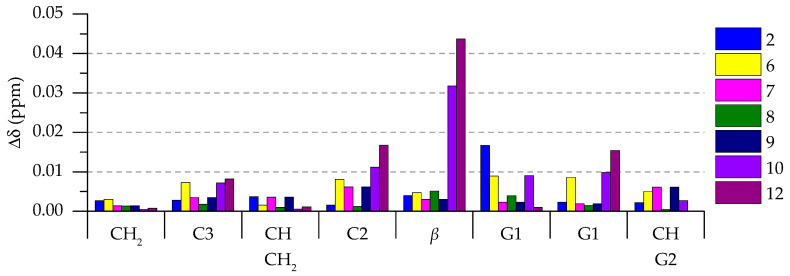
^1^H NMR chemical shift deltas (Δδ, ppm) of *E. coli* lipid extracts in the presence of vescalagin **2**, geraniin **6**, chebulagic acid **7**, chebulinic acid **8**, punicalagin **9**, oenothein B **10**, and oenothein A **12**. The tannin numbering refers to Figure 1 and lipid proton assignations refer to Figure 5. Values are presented as Δδ (*n* = 1).

Initially, the HR-MAS measurements were done as described in Section 3.3 for the whole tannin series **1**–**13**. Preliminary selections were made based on these results as to which tannins showed the highest levels of interaction established by the magnitude of the chemical shift changes seen in the ^1^H spectra (Figure 3 and Figure A1) as well as whether the aromatic protons of tannins showed any measurable correlations to the lipid protons in the NOESY spectra. From these tests, the highest level of lipid interaction was detected with tellimagrandin I **1**, casuarictin **3**, tellimagrandin II **4**, pentagalloylglucose **5**, sanguiin H-6 **11**, and lambertianin C **13**. The rest of the ellagitannins (**2**, **6**–**8**, **10**, and **12**) did not show detectable levels of correlation in the NOESY experiments, which is why the subsequently measured repetitions (*n* = 4) were made only to the aforementioned six tannins.

#### 2.2.1. H Chemical Shift Deltas of *E. coli* Lipid Extract in the Presence of Tannins

We determined how much the ^1^H signals of *E. coli* lipid extracts shifted in the presence of the added ellagitannins or pentagalloylglucose. The shifting of lipid signals is a result of the ring current effect from the aromatic ring structures of the ETs. The magnitude of the signal shift informed how much the added tannin had an effect on the spatial surrounding of the lipid proton, thus indicating how far into the lipid bilayer the tannin can penetrate [14]. Figure 3 shows the chemical shift delta values for the selected tannins **1**, **3**, **4**, **5**, **11**, and **13**. It seems that the chemical shifts of H-C2, H-*β*, and H-G1 were the most affected by the addition of the tannins. This indicates that the tannins can penetrate into the lipid bilayer at least until H-C2, but the following fatty acid chain beginning with H-C3 and the subsequent CH_2_-chain, H-CHCH_2_, and H-CH are not measurably affected.

Mainly, the monomeric ETs (**1**, **3** and **4**) and pentagalloylglucose (**5**) induced larger changes than the oligomeric ETs (**11** and **13**). Additionally, within the monomeric ETs, it seemed that the more hydrophobic ones cause more change in the chemical shifts of lipid protons than the less hydrophobic ones. Tellimagrandin II (**4**) caused the largest change in the lipid proton chemical shifts out of all the ETs. This was exceeded only by pentagalloylglucose (**5**) in regard to some of the lipid protons. These trends followed the assumption that more hydrophobic compounds would interact more with lipids, and thus penetrate more into the lipid bilayer structure. It is worth noting that the structural flexibility of the studied monomeric tannins increases in the same order as the hydrophobicity (**1**→**3**→**4**→**5**), and probably also contributes significantly to the rate of lipid interaction of the tannin. However, hydrophobicity alone does not determine whether an ET interacts measurably with *E. coli* or of the magnitude of the interaction. This can be seen from Appendix A Figure A1, where geraniin **6**, chebulagic acid **7**, and chebulinic acid **8** had almost no effect on the chemical shifts of the lipid protons, although all three of these ETs have been shown to be highly hydrophobic [18].

#### 2.2.2. NOESY Cross Relaxation Rates of the Aromatic Protons of Tannins

**Figure 4:**
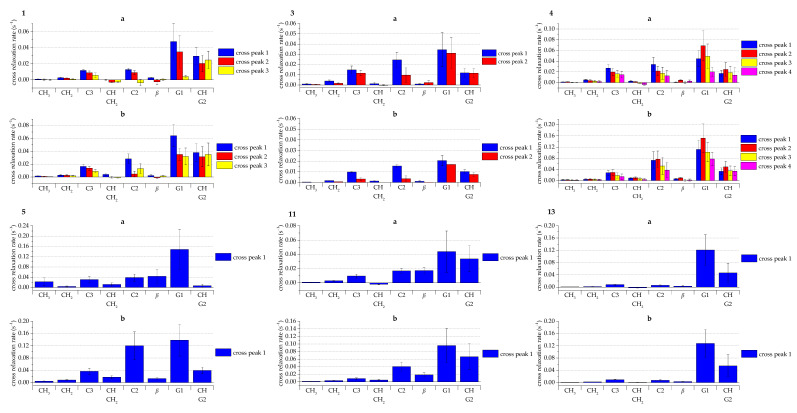
Cross relaxation rates of the aromatic protons (cross peaks 1–4 refer to Section 3.3 for definitions) of tellimagrandin I **1**, casuarictin **3**, tellimagrandin II **4**, pentagalloylglucose **5**, sanguiin H-6 **11**, and lambertianin C **13** against different lipid protons with mixing times of (**a**) 0.1 s and (**b**) 0.3 s. The tannin numbering refers to Figure 1 and lipid proton assignations refer to Figure 5. Cross peak labels are defined in Section 3.3. Values are presented as s^−1^ (average values and standard error, n = 4).

The NOESY experiment is particularly useful for determining if the ET and lipid are spatially close to each other, because typically, an NOE correlation is only detected when the correlating protons are spaced closer than 5 Å apart. NOESY cross relaxation rates for tellimagrandin I **1**, casuarictin **3**, tellimagrandin II **4**, pentagalloylglucose **5**, sanguiin H-6 **11**, and lambertianin C **13** were calculated based on Equation described in Section 3.3, and are presented in Figure 4 for the mixing times of 0.1 s (**a**) and 0.3 s (**b**). Theoretically, a higher cross relaxation rate indicates that the correlating protons are closer to each other, and thus correlate more intensely. The higher mixing time of 0.3 s proved to be more effective in reaching a higher measureable cross relaxation rate with the exception of **13**, which had equally high rates with both measured mixing times. This is probably because of the relatively high molecular weight of 2805.90 Da of **13**. Already with **11** having the molecular weight of 1871.27 Da, the difference between mixing times was not as distinct as with the smaller tannins.

Measured tannins showed the highest cross relaxation rates against lipid protons H-C3, H-C2, H-G1, and H-CH/H-G2, which again would indicate that they effectively penetrate into the lipid bilayer at least enough to correlate with the lipid protons closer to the lipid headgroups. From these protons, the most prominent cross relaxation was measured against H-G1 for all the tannins. Tellimagrandin II **4** and pentagalloylglucose **5** showed the highest cross relaxation rates out of the selected tannins, which is most likely due to their rather flexible structures caused by the many freely rotating galloyl groups and relatively high hydrophobicity, enabling them to enter the lipid bilayer structure effectively. ETs **1** and **3** exhibited measurable cross relaxation rates, but they were the lowest out of the selected tannins, which is probably due to their reasonably rigid structures when compared with **4** and **5**. The larger ETs dimeric **11** and trimeric **13** displayed moderate cross relaxation rates, with the trimeric **13** being almost as high as the most effective monomers. Both of the ET oligomers that showed NOE correlations with the lipid protons had multiple free galloyl groups. In comparison, dimeric oenothein B **10** and trimeric oenothein A **12** did not display any measurable NOE correlations. Both **10** and **12** have only one free galloyl group per monomeric unit and a macrocyclic structure caused by two oligomeric linkages between the monomeric units, which makes their structures rigid. Similarly, vescalagin **1** and punicalagin **9** did not show NOE correlations with the lipid, which is explained by their rigid structures, with the former having an HHDP group and an NHTP group, while the latter has an HHDP group and a gallagyl group. However, the lack of correlation from geraniin **6**, chebulagic acid **7**, and chebulinic acid **8** was surprising because they have been shown to be rather hydrophobic and, additionally, all three of them have varying amounts of free galloyl groups in their structure, making them moderately flexible. It might be that the DHHDP group of **6** and the chebuloyl group of **7** and **8** make them less likely to penetrate into the lipid bilayer when compared with the other monomeric tannins (**1**, **3**, **4**, and **5**) shown to interact with the lipid protons.

For the monomeric ETs **1**, **3**, and **4**, cross relaxation rates could be calculated separately for some of the aromatic protons, which are defined in Section 3.3. The differences in the cross relaxation rates between these aromatic protons informs us how the studied ET is oriented in the lipid bilayer. It seems that the cross relaxation rates of the cross peaks correlating from the ETs’ galloyl group protons (**1** cross peak 1, **3** cross peak 1, **4** cross peaks 1 and 2) are higher than the cross peaks from the ETs’ HHDP group protons (**1** cross peaks 1 and 2, **3** cross peak 2, **4** cross peaks 3 and 4), which suggests that the galloyl groups of ETs are oriented more towards the lipid than their HHDP groups.

#### 2.2.3. Effect of ET Concentration on the ET–Lipid Interaction

**Figure A2:**
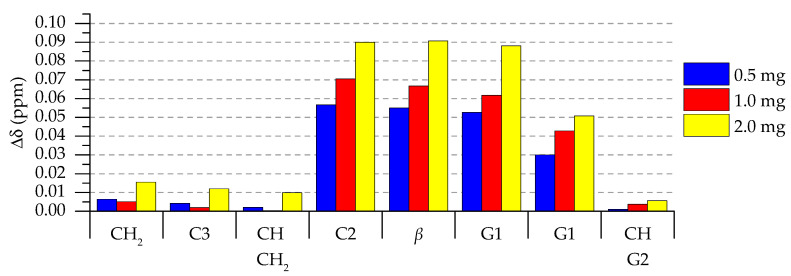
^1^H NMR chemical shift deltas (Δδ, ppm) of *E. coli* lipid extracts in the presence of three different weighed amounts of tellimagrandin II **4**.

**Figure A3:**
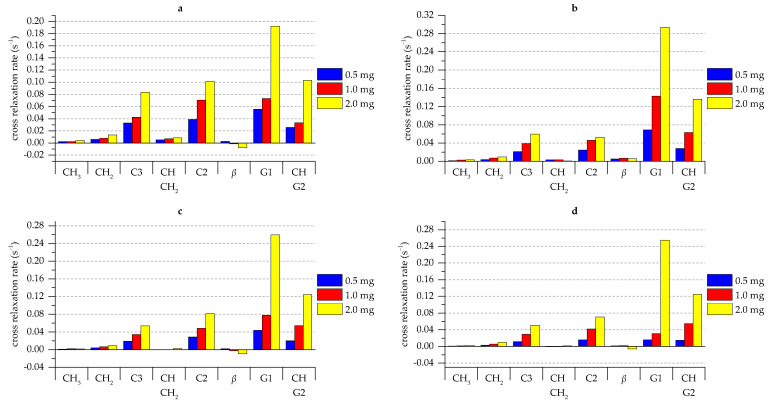
Cross relaxation rates of the aromatic protons (cross peak 1 (**a**), cross peak 2 (**b**), cross peak 3 (**c**), and cross peak 4 (**d**)) of three different weighed amounts of tellimagrandin II **4** against different lipid protons with a mixing time of 0.1 s. The lipid proton assignations refer to Figure 5 and cross peak labels are defined in Section 3.3.

**Figure A4:**
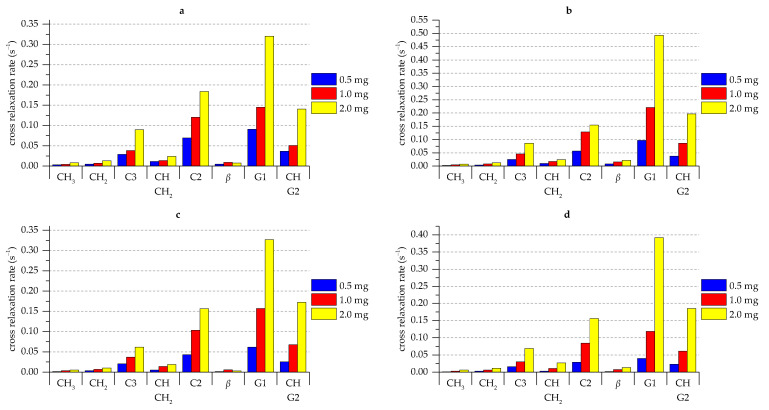
Cross relaxation rates of the aromatic protons (cross peak 1 (**a**), cross peak 2 (**b**), cross peak 3 (**c**), and cross peak 4 (**d**)) of three different weighed amounts of tellimagrandin II **4** against different lipid protons with a mixing time of 0.3 s. The lipid proton assignations refer to Figure 5 and cross peak labels are defined in Section 3.3.

The effect of concentration of the added tannins was tested with tellimagrandin II **4** while keeping the amount of *E. coli* lipid extract constant (4.0 mg). The weighed amounts of **4** were 0.5 mg, 1.0 mg, and 2.0 mg. Appendix B Figure A2 shows the chemical shift deltas of the lipid extracts protons in the presence of the different amounts of **4** in the solution. Appendix B Figure A3 and Figure A4 show the cross relaxation rates of aromatic protons of **4** against different lipid protons in the same three concentrations. Both the chemical shift changes of the lipid protons and the cross relaxation rates of aromatic protons of **4** against lipid protons increase when the concentration of **4** increases. However, it is noteworthy that the increase in cross relaxation rates when moving from 1.0 mg to 2.0 mg is drastically larger than when moving from 0.5 to 1.0 mg, so the concentration effect might not be directly linear.

#### 2.2.4. Effect of Lipid Batch on the ET–Lipid Interaction

We used three different batches of the lipid extract and performed the replicate HR-MAS measurements in order to account for the possible biological variability in the lipid extract. ^1^H HR-MAS NMR spectra of these different *E*. *coli* extract batches (a–c) measured in D_2_O at 25 °C are shown in Figure S2 in Supplementary Materials. We noticed that the interaction between ETs and lipids was the strongest in those lipid extracts that showed less additional unassigned peaks in the ^1^H spectrum (Figure S2a,c). Most probably, the unknown component in the lipid extract (Figure S2b) also interacted with ETs and affected the interactions between ETs and lipids.

### 2.3. Analysis of Stability of ET–Lipid Solution with UPLC-DAD-MS

The stability of the ellagitannins was monitored to verify that no metabolites or degradation products were formed in the solution with the lipid extract and that all the detected interactions were caused by the original ET. Sample preparation and measurements were performed as described in Section 3.4. The samples were made in H_2_O to resemble the conditions in which the HR-MAS NMR measurements were done, but some of the studied ETs and pentagalloylglucose are so hydrophobic that some decline in solubility was expected during the stability measurements. During the 40 h analysis, only traces of ET metabolite products were detected and example chromatograms of the analysis of tellimagrandin II (**4**) are shown in Figure S1 in Supplementary Materials. This confirmed that ETs were stable in the conditions used and that the detected interaction was indeed caused by the studied initial ET.

## 3. Materials and Methods

### 3.1. Chemicals

Technical grade acetone was purchased from VWR (Haasrode, Belgium). Analytical grade, analytical grade methanol, HPLC gradient grade acetonitrile, HPLC gradient grade methanol, HPLC grade formic acid, HPLC grade phosphoric acid, and LC-MS grade formic acid were purchased from VWR International (Fontenay-Sous-Bois, Paris, France). LC-MS grade acetonitrile was purchased from Merck (KGaA, Darmstadt, Germany). D_2_O (99.90% purity), MeOD-*d*4 (99.80% purity, water < 0.03%), and acetone-*d*6 (99.80% purity, water < 0.02%) were purchased from Eurisotop, a subsidiary of Cambridge Isotope Laboratories, Inc. (Tewksbury, MA, USA). Ultra-pure type I water was purified with Merck Millipore Synergy UV system.

**Figure 5:**
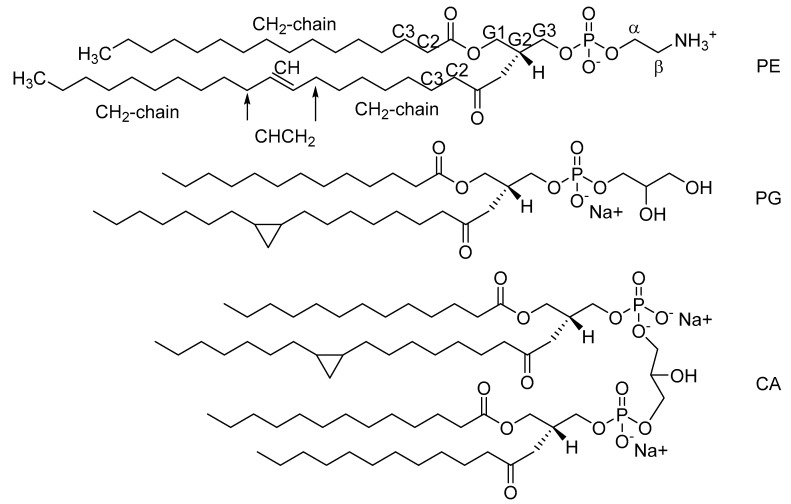
Example structures of the known components of the commercial *E. coli* lipid extract, L-*α*-phosphatidylethanolamine (PE), L-*α*-phosphatidylglycerol (PG), and cardiolipin (CA), with labeling on the PE lipid to illustrate the NMR assigned protons. The length and precise structure of the fatty acid chains (CH_2_) have not been determined.

Commercial *E. coli* lipid extract was purchased from Avanti Polar Lipids (Alabaster, AL, USA). *E. coli* extract contained L-*α*-phosphatidylethanolamine (PE, 57.5 w-%), L-*α*-phosphatidylglycerol (PG, 15.1 w-%), and cardiolipin (CA, 9.8 w-%), and the remaining 17.6 w-% consisted of an unidentified lipid, according to the manufacturer. Figure 5 shows the representative structures of PE, PG, and CA lipids in the extract mixture along with labels for all the protons that were assigned shown in the PE lipid as examples. Because of the unknown component of the lipid extract, we purchased three different batches of the lipid extract with which we performed the replicate HR-MAS measurements in order to account for the possible biological variability in the lipid extract. ^1^H HR-MAS NMR spectra of the different batches used in the study are shown in Figure S2 in Supplementary Materials. Pure PE lipid was purchased from Avanti Polar Lipids (Alabaster, AL, USA).

### 3.2. Isolation of Ellagitannins and Pentagalloylglucose

The purification of the selected ETs and pentagalloylglucose followed our previously reported extraction and isolation methodology, which briefly goes as follows: extraction, Sephadex LH-20 funnel and column chromatography, and preparative and semipreparative HPLC [5,6,8,10,16,19,20]. Purified tannins were characterized based on their retention times in reverse-phase LC, UV spectra, molecular ions, and characteristic fragments established in our previous work [5]. Monomeric tannins tellimagrandin I (**1**) and tellimagrandin II (**4**) were isolated from meadowsweet inflorescence; vescalagin (**2**) from the flowers and leaves of purple loosestrife; casuarictin (**3**) from sea buckthorn leaves; pentagalloylglucose (**5**) from tannic acid purchased from J.T. Baker (Denventer, Holland); and geraniin (**6**), chebulagic acid (**7**), chebulinic acid (**8**), and punicalagin (**9**) from *Terminalia chebula* fruits. Oligomeric ellagitannins oenothein B (**10**) and oenothein A (**12**) were isolated from willow herb flowers and sanguiin H-6 (**11**) and lambertianin C (**13**) from raspberry leaves. The stereochemistries of acyclic ellagitannins vescalagin (**2**) and castalagin were reinvestigated by Matsuo et al. 2015, showing that the NHTP-group exists in (S,R) configuration and vescalagin **2** is presented according to this revision [21]. ^1^H NMR spectra of compounds 1–13 are shown in Figures S3–S15 in Supplementary Materials.

### 3.3. NMR Analyses

NMR measurements were performed with either a Bruker Avance-III spectrometer equipped with a Prodigy TCI (inverted CryoProbe) cooled via liquid nitrogen, which was operated at 600.16 MHz for ^1^H and 125.76 MHz for ^13^C or a Bruker Avance-III spectrometer equipped with a high-resolution magic angle spinning (HR-MAS) probe, which was operated at 399.75 MHz for ^1^H and 100.52 MHz for ^13^C. Typical ^1^H and ^13^C spectra were recorded in addition to multiple 2D spectra including COSY (correlation spectroscopy), NOESY (nuclear Overhauser effect spectroscopy), HSQC (heteronuclear single quantum coherence), and HMBC (heteronuclear multiple bond correlation). Measurements were done at 25 °C in either MeOD-*d*4 or D_2_O.

The following sample preparation method was adapted from Grélard et al., 2010 [22]. For HR-MAS measurements, 4.0 mg of *E. coli* lipid extract was weighed into an eppendorf along with 1.0, 2.0, or 3.0 mg of the studied tannin. The amount of tannin utilized depended on the degree of oligomerisation of tannins; 1.0 mg was used for monomeric tannins (1–9), 2.0 mg for dimeric tannins (10–11), and 3.0 mg for trimeric tannins (12–13). The lipid extract/tannin mixture was dissolved in 100 µL of D_2_O and subsequently handled via a freeze–thaw method in order for the lipids to form a bilayer [22]. The method consisted of shaking the sample vigorously in room temperature, freezing it in liquid nitrogen, and heating it in a warm water bath. This cycle was then repeated four times until a hazy emulsion was formed. The emulsion was transferred into an HR-MAS insert (50 µL, Bruker), which was subsequently placed into a ZrO_2_ HR-MAS rotor (4 mm, Bruker). The rotor was placed in the instrument, the MAS unit was operated at 9 kHz rotational speed, and the temperature was set to 25 °C. A typical measurement set consisted of a standard ^1^H experiment with water presaturation (zgpr), followed by two NOESY experiments with mixing times of 0.1 s and 0.3 s, and finally a second ^1^H experiment with water presaturation.

During HR-MAS NMR data processing, the ^1^H spectra were calibrated based on the CH_3_ peaks value (δ = 0.9445 ppm) for all the tannin samples as well as the referenced pure lipid sample against which the chemical shift deltas were calculated. The CH_3_ peak was used as a reference as the methyl group end of the fatty acid chain of the lipids (Figure 5) resides deepest in the formed lipid bilayer structure, and should thus be the least exposed to the tannins’ influence, and the solvent signal of D_2_O was suppressed and thus unusable. Cross relaxation rates were calculated from the NOESY spectra based on the following equation [15]:

$$\mathrm{cross}\;\mathrm{relaxation}\;\mathrm{rate}=\frac{\left(\frac{\mathrm{cross}\;\mathrm{peak}\;\mathrm{volume}}{\mathrm{number}\;\mathrm{of}\;\mathrm{cross}\;\mathrm{peak}\;\mathrm{protons}}\right)\;}{\mathrm{diagonal}\;\mathrm{peak}\;\mathrm{volume}*\mathrm{mixing}\;\mathrm{time}}$$

From the NOESY spectra, lipid signal volumes (diagonal peak volume, abs) and their correlation signals to the tannins’ aromatic protons (cross peak volumes, abs) were integrated for both used mixing times (0.1 s and 0.3 s). For tellimagrandin I **1**, casuarictin **3**, and tellimagrandin II **4**, the cross peaks could be integrated individually for some of the aromatic protons because the aromatic protons could be identified and were resolved enough in the ^1^H spectra, and they are labeled in Figure 4 as follows. For tellimagrandin I, cross peak 1 refers to the protons of galloyl group attached to O2 and O3 of glucose, cross peak 2 to the proton of HHDP group attached to O6 of glucose, and cross peak 3 to the proton of HHDP group attached to O4 of glucose. For casuarictin, cross peak 1 refers to the proton of galloyl group attached to O1 of glucose and cross peak 2 to the protons of HHDP group attached to O2, O3, O4, and O6 of glucose. For, tellimagrandin II cross peak 1 refers to the proton of galloyl group attached to O1 of glucose, cross peak 2 to the protons of galloyl group attached to O2 and O3 of glucose, cross peak 3 to the proton of HHDP group attached to O6 of glucose, and cross peak 4 to the proton of HHDP attached to O4 of glucose. For pentagalloylglucose **5**, sanguin H-6 **11**, and lambertianin C **13**, the aromatic protons could not be separated based on the resolution achieved with the 400 MHz HR-MAS instrument, so the cross relaxation rates were calculated for all the aromatic protons as a group and labeled as cross peak 1.

### 3.4. UPLC-DAD-MS Analyses

The UPLC-DAD used in all analyses was an Acquity UPLC (Waters Corp., Milford, MA, USA) instrument consisting of a binary solvent manager, a column, and a diode array detector. The column utilized was an Aquity BEH phenyl column (2.1 × 100 mm, 1.7 µm; Waters Corp., Wexford, Ireland). The mobile phase consisted of acetonitrile (A) and 0.1% aqueous formic acid (B) with a constant flow rate of 0.5 mL min^−1^ with the following gradient: 0–0.5 min: 0.1% A; 0.5–5.0 min: 0.1–30% A (linear gradient); 5.0–5.1 min: 30–90% A (linear gradient); 5.1–7.1 min: 90% A; 7.1–7.2 min: 90–0.1% A (linear gradient); and 7.2–8.5 min: 0.1 % A. Column temperature was 25 °C. The UPLC was connected to a Q Exactive hybrid quadrupole-Orbitrap mass spectrometer (Thermo Fisher Scientific GmbH, Bremen, Germany) via a heated ESI (electrospray ionization) source. Sheath gas and auxiliary gas flow rates were set to 60 Au and 20 Au, respectively, and spray voltage was set to 3.0 kV. The DAD detector was set to collect UV-data from 190–500 nm and the Orbitrap was operated with full scan at a mass range of 150–2000 Da with a resolution of 70,000.

For the stability measurements, 0.5 mg of tannin was weighed together with 2 mg of the *E. coli* lipid extract in an eppendorf and dissolved in 2.5 mL of H_2_O. A fivefold dilution of this sample was made and filtered through a 0.2 µM filter (PTFE). This sample was injected hourly for 40 h to the UPLC-DAD-MS instrument. After the 20th injection, a new sample was filtered from the originally prepared tannin–lipid mixture to account for the effect that the filtration might have had on the sample.

### 3.5. Data Analysis and Software

UPLC measurements and quantitations were done with Thermo Xcalibur version 4.1.31.9 (Thermo Fisher Scientific Inc., Waltham, MA, USA). NMR data were measured and analyzed using TopSpin software versions 3.5 pl 7 and 3.5 pl 5 (Bruker, Billerica, MA, USA). Graph visualizations were done with Origin 2016 (64-bit) software version SR2 b9.3.2.303 (OriginLab, Northampton, MA, USA).

## 4. Conclusions

The main aim of this study was to uncover if the possible interactions between ellagitannins and lipids can be detected and studied with the help of HR-MAS NMR. The results showed that some ETs were more inclined to penetrate into the lipid bilayer than other ETs. The main deciding structural factors based on the results of the ETs studied here were that ETs with a cyclic polyol and high structural flexibility are more likely to interact with lipids. This result is well in line with the studied hydrophobicity of these compounds, i.e., more hydrophobic ETs were shown to interact more with lipids. Most of the detected interaction seemed to happen towards the headgroups of the lipids and less with the fatty acid chain. As a technique, HR-MAS NMR proved to be very suitable for the study of the ET–lipid mixtures owing to the fact that it tolerates the sort of semisolid emulsions these compounds form in an aqueous measurement environment.

## Data Availability

The data presented in this study are available on request from the corresponding author.

## Supplementary Materials

The following materials are available online. Figure S1: UPLC-DAD chromatograms at 280 nm from stability measurements. Figure S2: 1H HR-MAS NMR spectra of the different E. coli extract batches used in this study. Figures S3–S15: 1H NMR spectrum of compounds 1–13.
