# Supplementary material for: Ellagitannin–Lipid Interaction by HR-MAS NMR Spectroscopy
Source: Molecules. 2021 Jan 12;26(2):373. doi: 10.3390/molecules26020373 (PMC7828275; doi:10.3390/molecules26020373)
Supplement: Supplementary file 1 [file molecules-26-00373-s001.zip › Ellagitannin-lipid Interaction by HR-MAS NMR Spectroscopy - Supplementary Materials.docx]

Supplementary Materials

Ellagitannin-Lipid Interaction by HR-MAS NMR Spectroscopy

Valtteri Virtanen *, Susanna Räikkönen, Elina Puljula and Maarit Karonen

^1^ Natural Chemistry Research Group, Department of Chemistry, University of Turku, Turku FI-20014, Finland; [vtjvir@utu.fi](mailto:vtjvir@utu.fi) (V.V.); [susanna.raikkonen@outlook.com](mailto:susanna.raikkonen@outlook.com) (S.R.); [elina.puljula@gmail.com](mailto:elina.puljula@gmail.com) (E.P); [maarit.karonen@utu.fi](mailto:maarit.karonen@utu.fi) (M.K.)

***** Correspondence: vtjvir@utu.fi; Tel.: Tel.: +358-29-450-3205 (V.V.)

Content

**Figure S1.** UPLC-DAD chromatograms at 280 nm for tellimagrandin II **4** and *E. coli* mixtures analyzed at **1**-, **10**-, **20**-, **30**- and **40**-hour time points displaying the whole intensity range (**a**) and a zoomed up intensity range (**b**) with traces of metabolites showing at the later time points.

**Figure S2.** ^1^H HR-MAS NMR spectra of the different *E. coli* extract batches (**a**-**c**) used in this study measured in D_2_O at 25 ℃.

**Figure S3.** ^1^H NMR spectrum of tellimagrandin I **1** measured in acetone-*d6* at 25℃.

**Figure S4.** ^1^H NMR spectrum of vescalagin **2** measured in acetone-*d6* at 25℃.

**Figure S5.** ^1^H NMR spectrum of casuarictin **3** measured in acetone-*d6* at 25℃.

**Figure S6.** ^1^H NMR spectrum of tellimagrandin II **4** measured in acetone-*d6* at 25℃.

**Figure S7.** ^1^H NMR spectrum of pentagalloylglucose **5** measured in acetone-*d6* at 25℃.

**Figure S8.** ^1^H NMR spectrum of geraniin **6** measured in acetone-*d6* at 25℃.

**Figure S9.** ^1^H NMR spectrum of chebulagic acid **7** measured in acetone-*d6* at 25℃.

**Figure S10.** ^1^H NMR spectrum of chebulinic acid **8** measured in acetone-*d6* at 25℃.

**Figure S11.** ^1^H NMR spectrum of punicalagin **9** measured in acetone-*d6* at 25℃.

**Figure S12.** ^1^H NMR spectrum of oenothein B **10** measured in acetone-*d6* at 25℃.

**Figure S13.** ^1^H NMR spectrum of sanguiin H-6 **11** measured in acetone-*d6* at 25℃.

**Figure S14.** ^1^H NMR spectrum of oenothein A **12** measured in acetone-*d6* at 25℃.

**Figure S15.** ^1^H NMR spectrum of lambertianin C **13** measured in acetone-*d6* at 25℃.





**Figure S1.** UPLC-DAD chromatograms at 280 nm for tellimagrandin II **4** and *E. coli* mixtures analyzed at **1**-, **10**-, **20**-, **30**- and **40**-hour time points displaying the whole intensity range (**a**) and a zoomed up intensity range (**b**) with traces of metabolites showing at the later time points eluting before **4**.


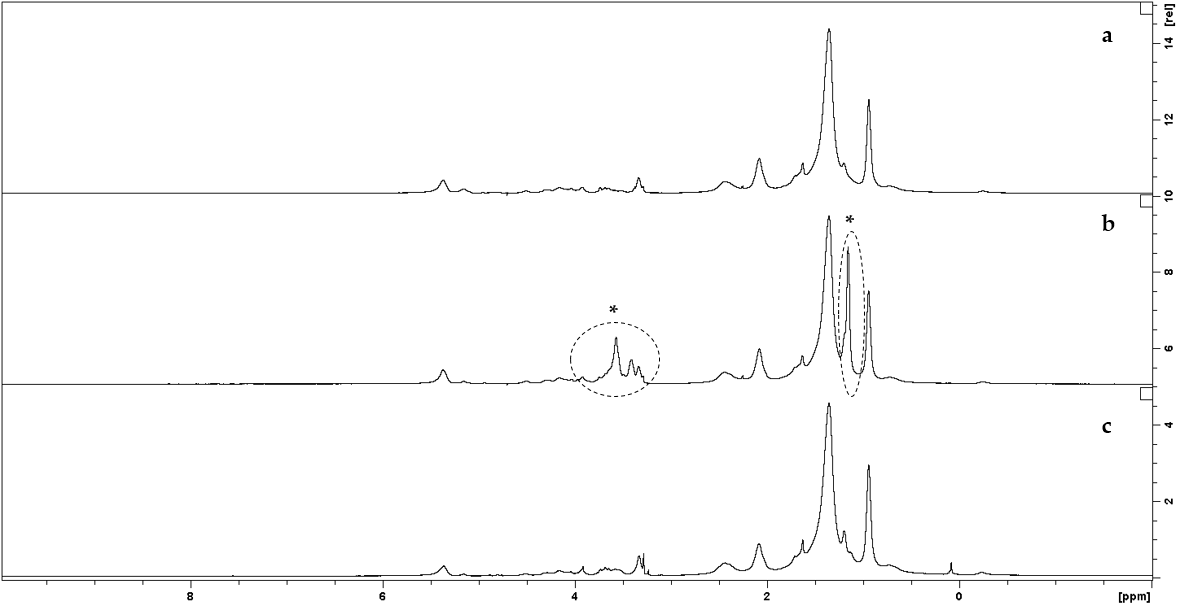


**Figure S2.** ^1^H HR-MAS NMR spectra of the different *E. coli* extract batches (**a**-**c**) used in this study measured in D_2_O at 25 ℃. *Varying unknown components highlighted in **b**.


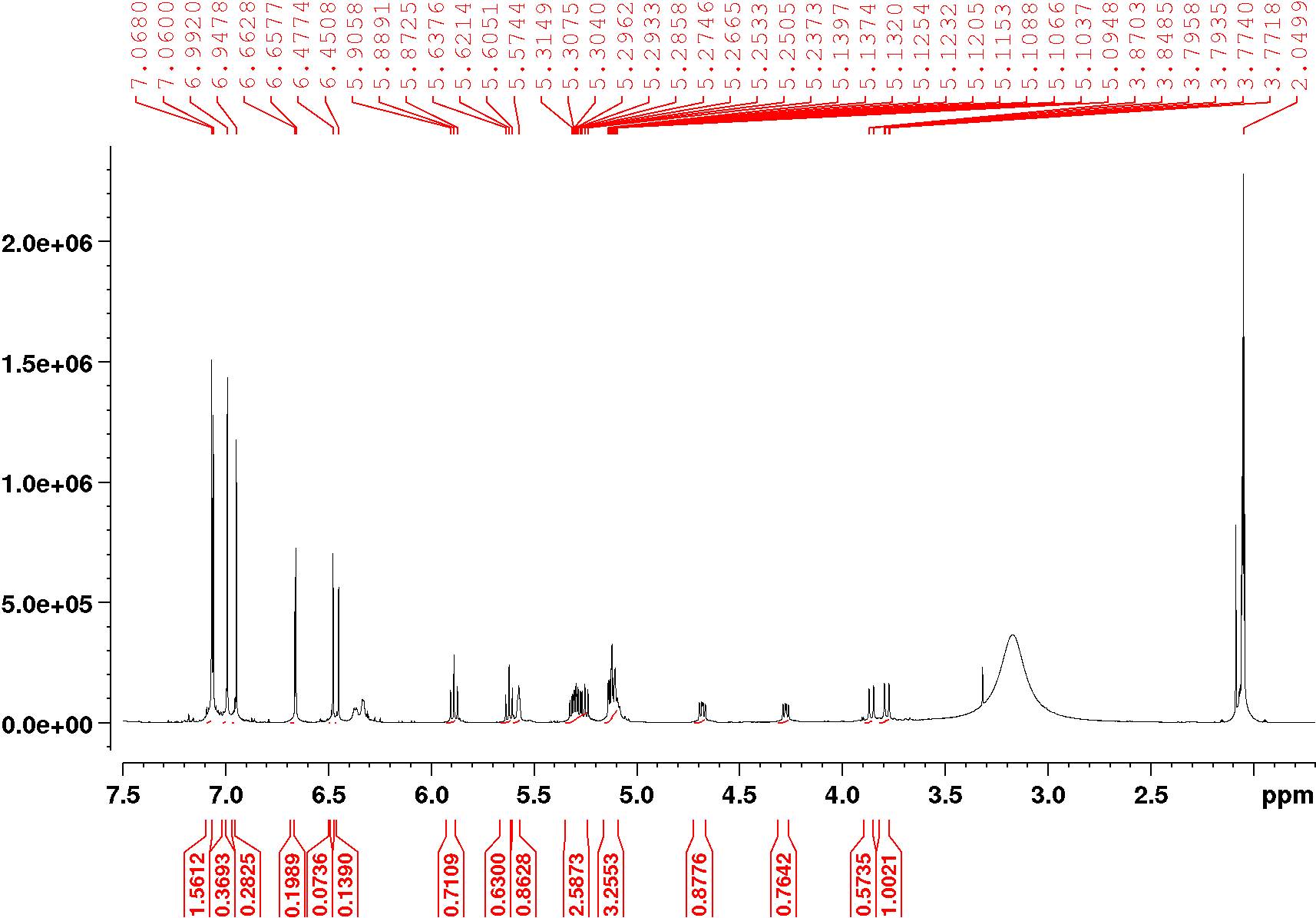


**Figure S3.** ^1^H NMR spectrum of tellimagrandin I **1** measured in acetone-*d6* at 25℃.


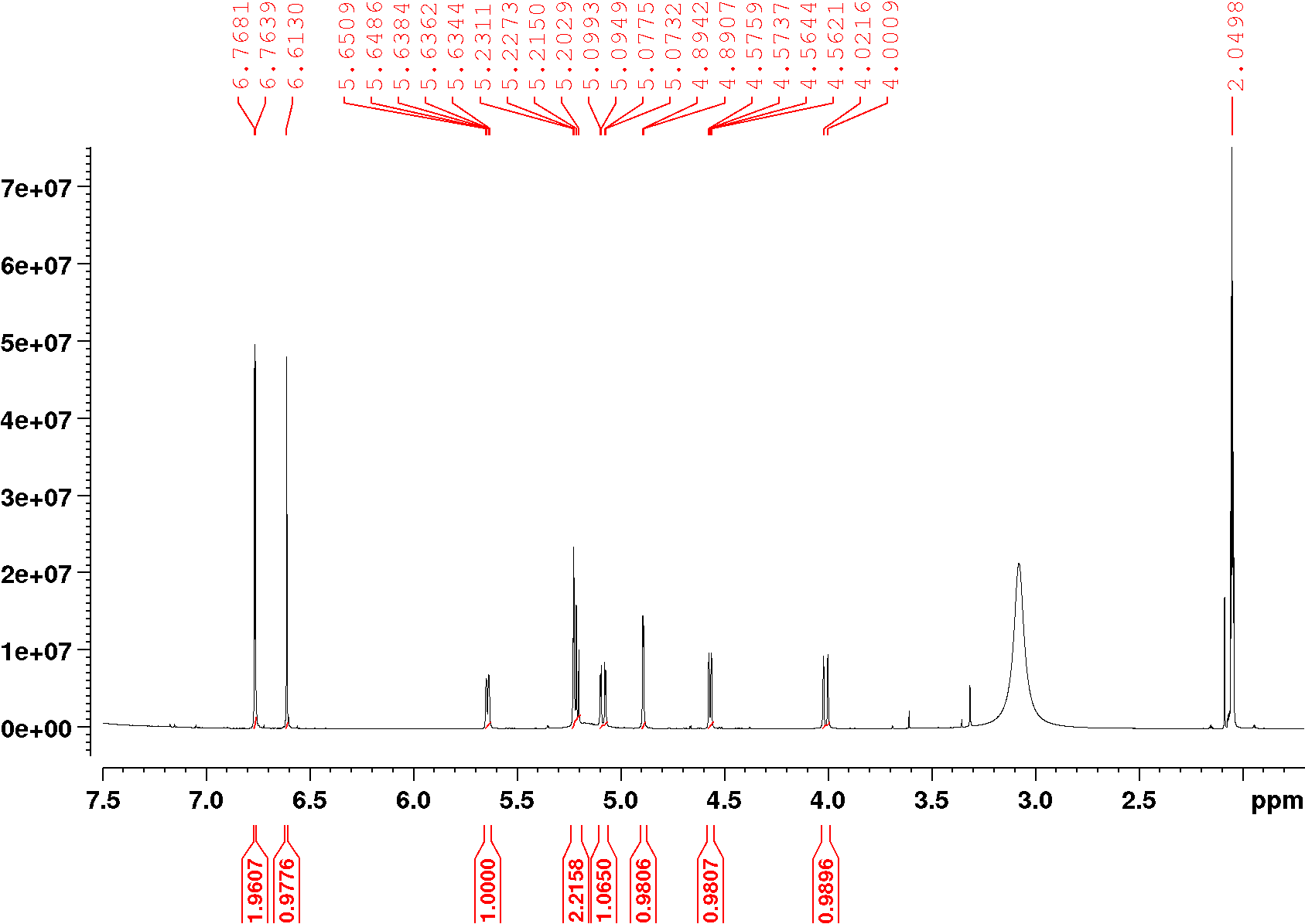


**Figure S4.** ^1^H NMR spectrum of vescalagin **2** measured in acetone-*d6* at 25℃.


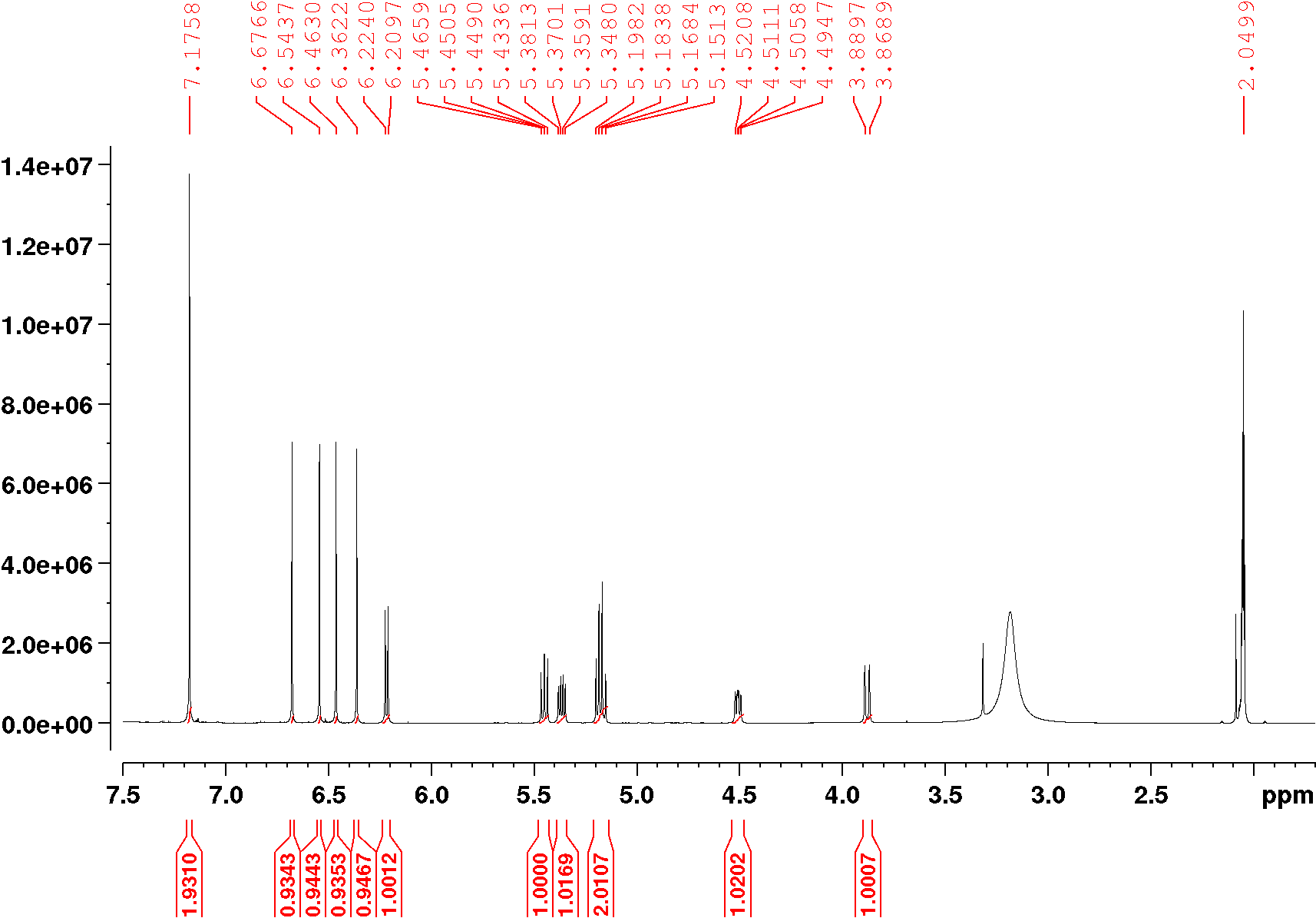


**Figure S5.** ^1^H NMR spectrum of casuarictin **3** measured in acetone-*d6* at 25℃.


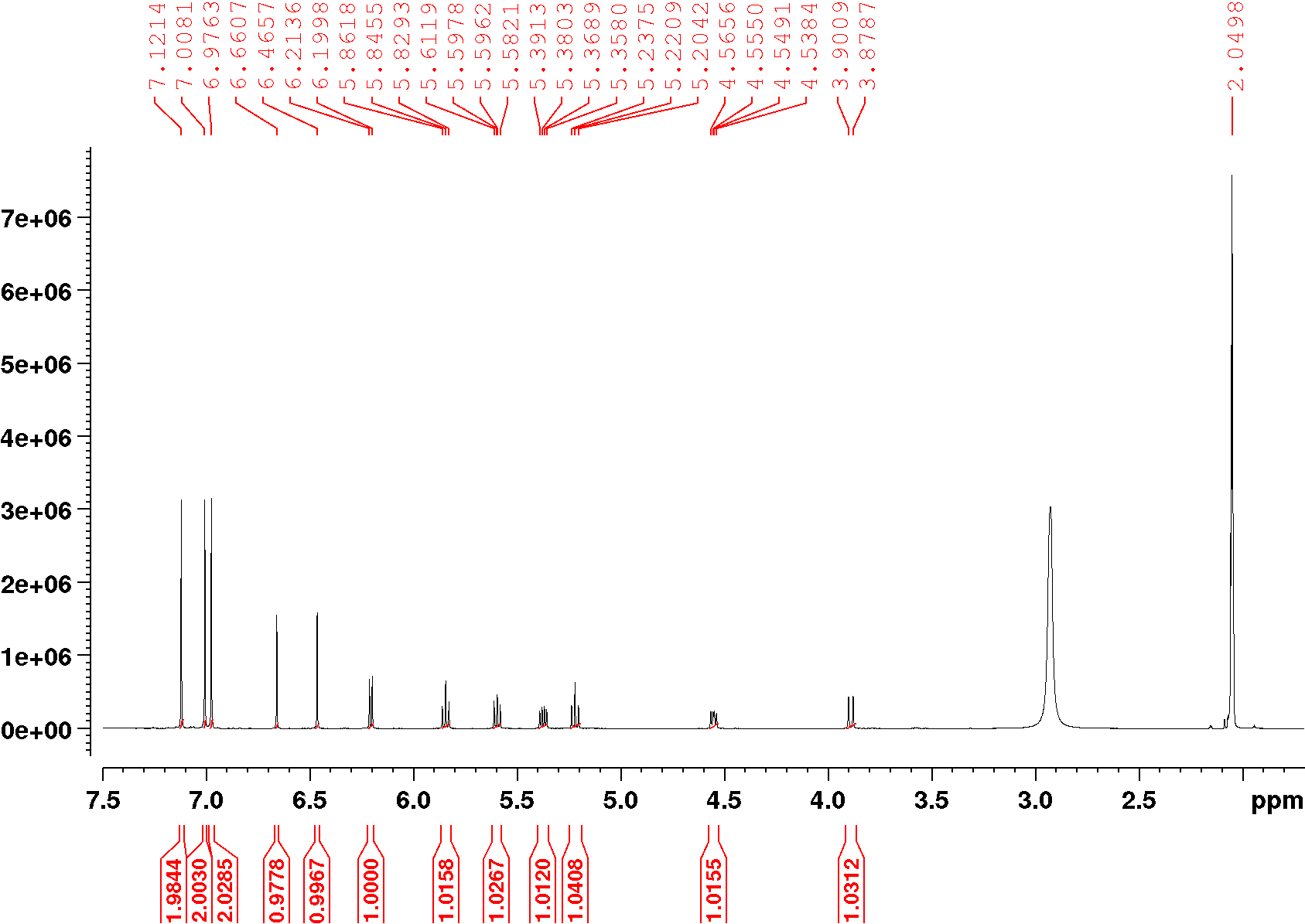


**Figure S6.** ^1^H NMR spectrum of tellimagrandin II **4** measured in acetone-*d6* at 25℃.


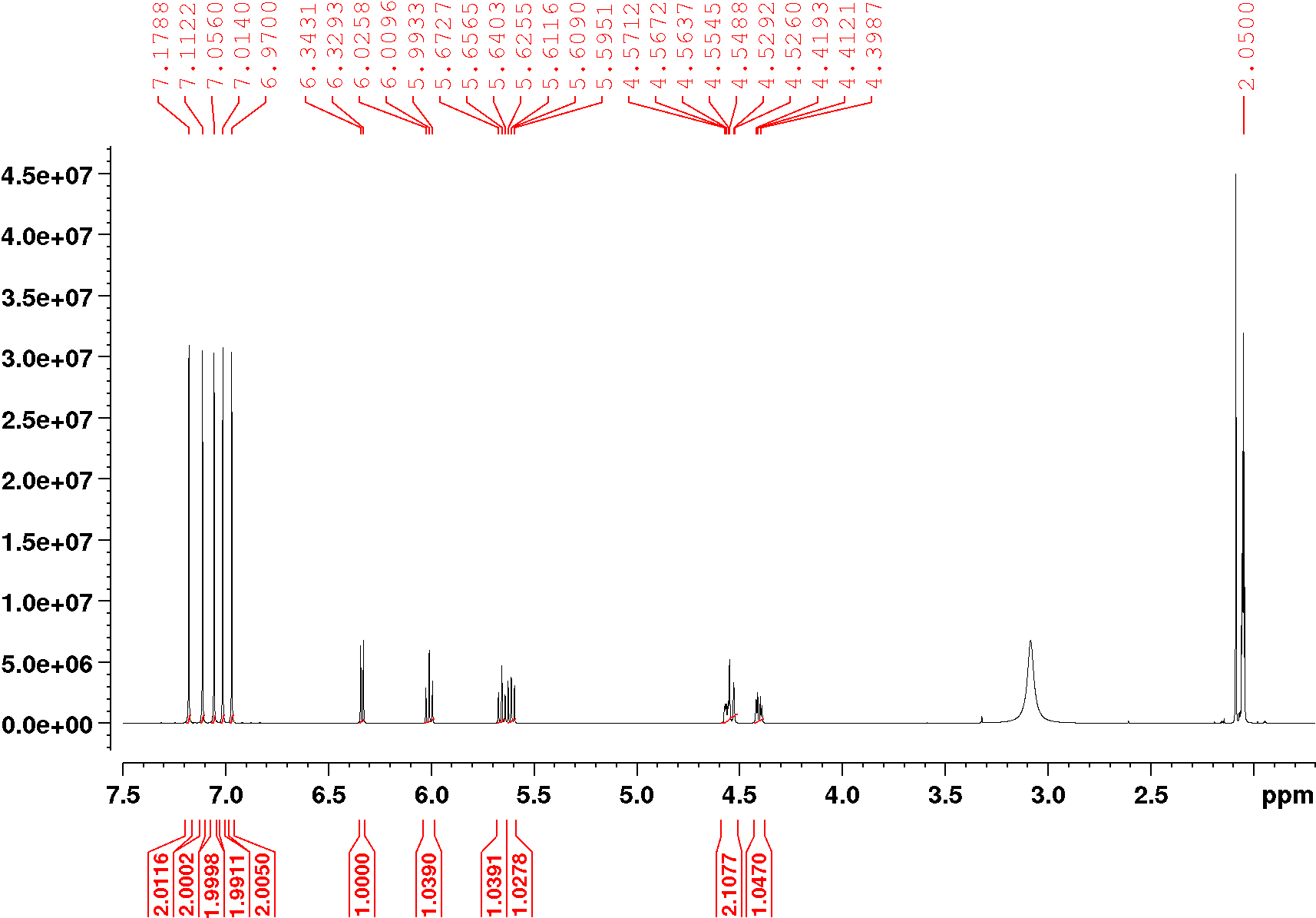


**Figure S7.** ^1^H NMR spectrum of pentagalloylglucose **5** measured in acetone-*d6* at 25℃.


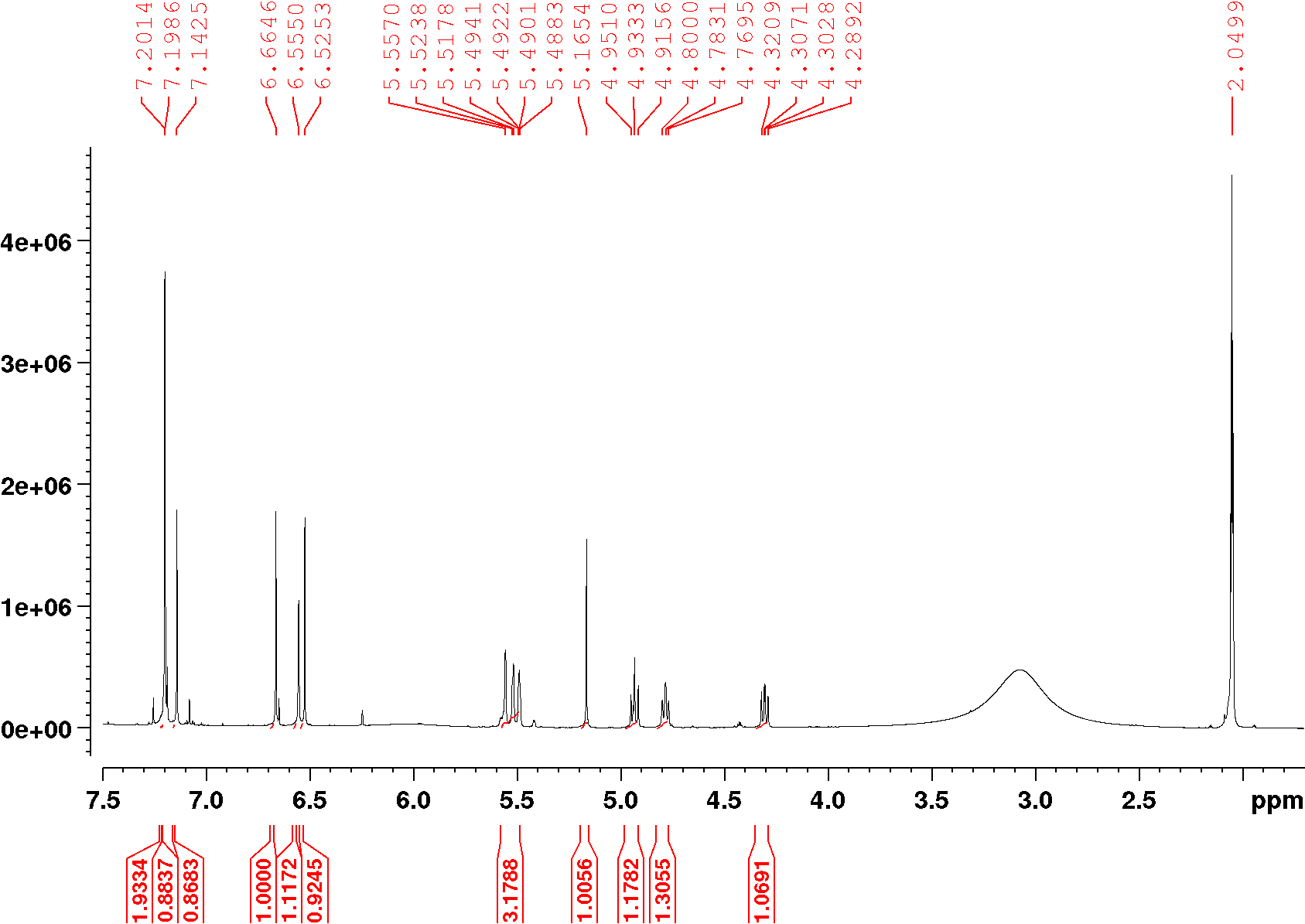


**Figure S8.** ^1^H NMR spectrum of Geraniin **6** measured in acetone-*d6* at 25℃.


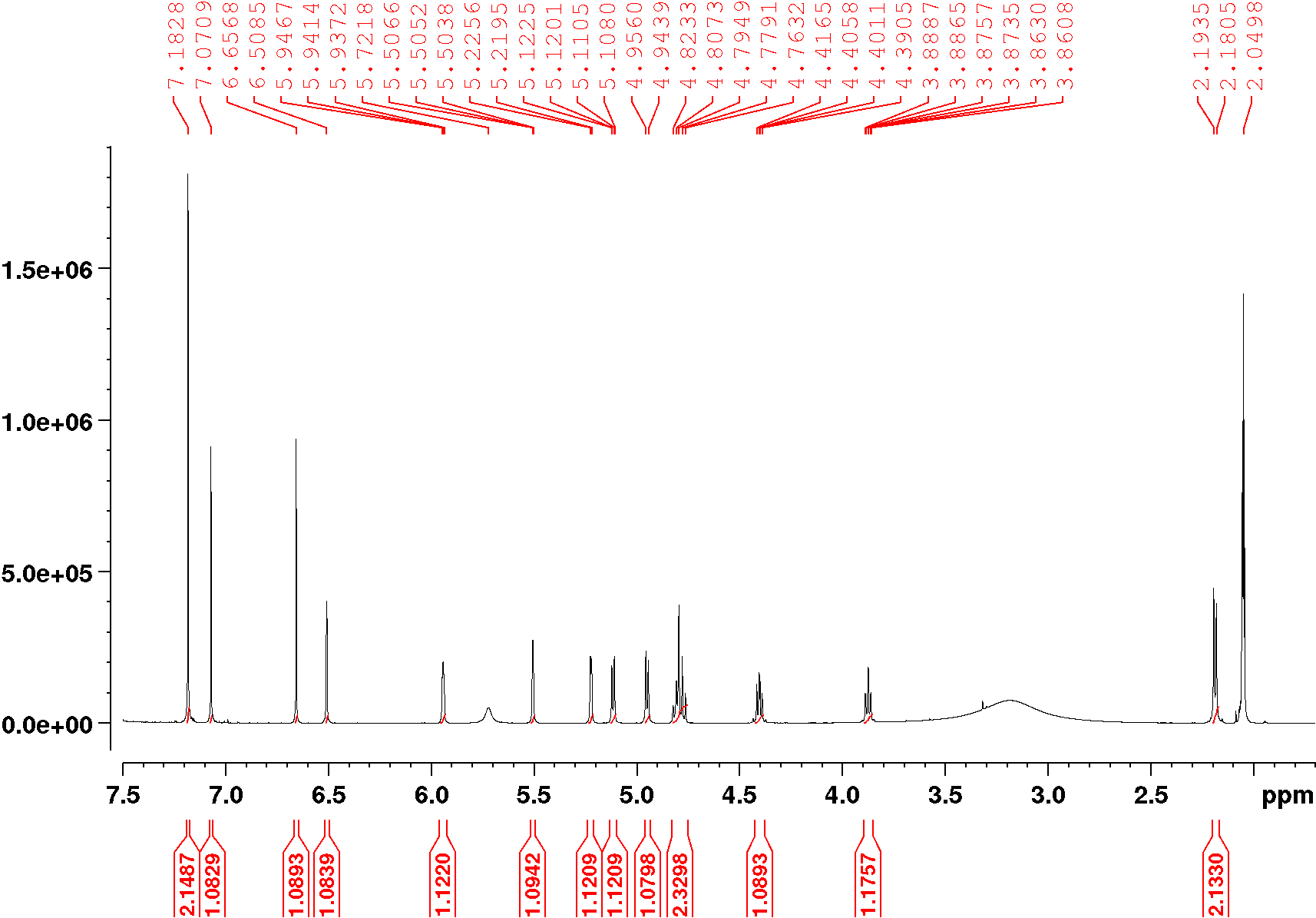


**Figure S9.** ^1^H NMR spectrum of chebulagic acid **7** measured in acetone-*d6* at 25℃.


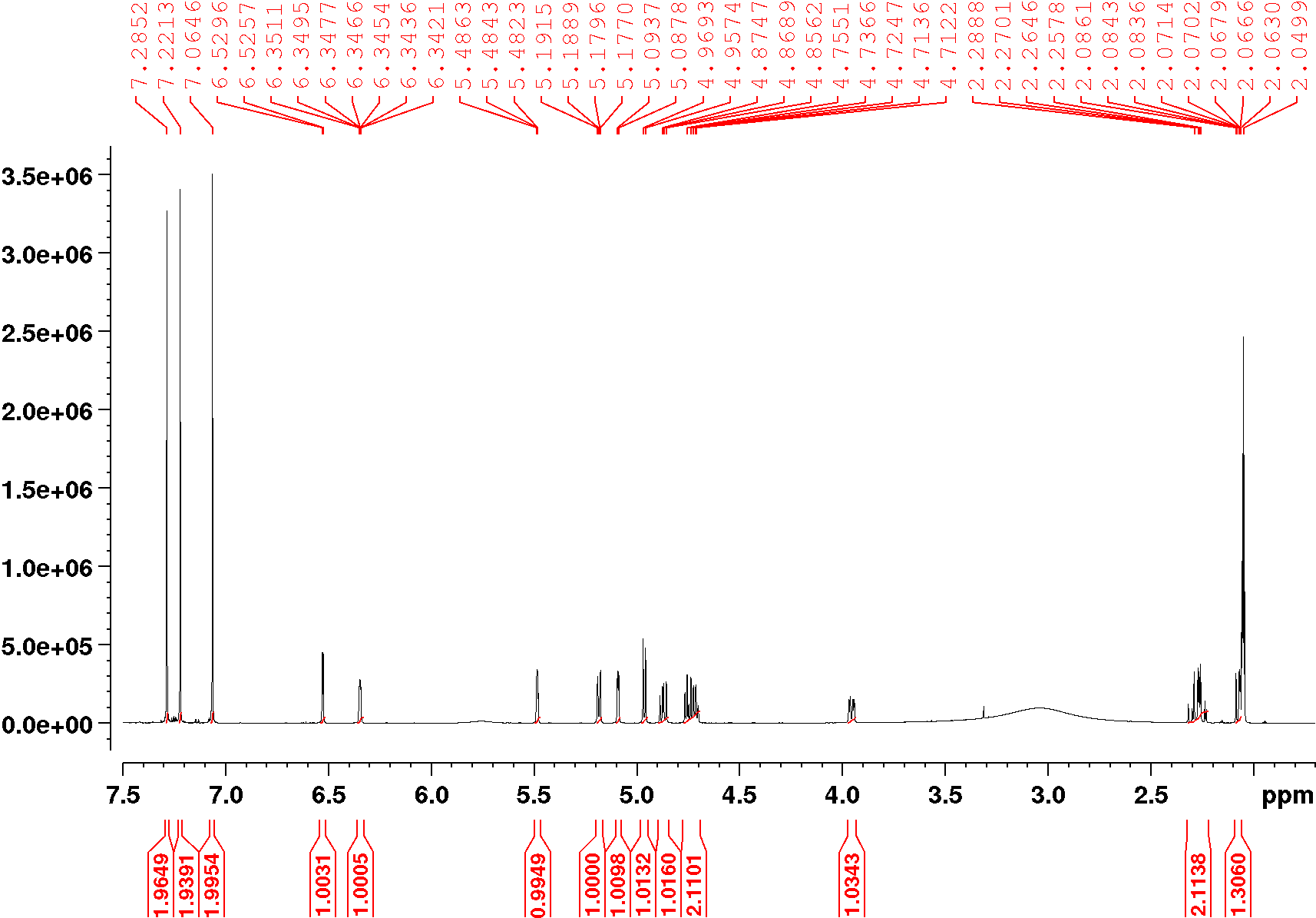


**Figure S10.** ^1^H NMR spectrum of chebulinic acid **8** measured in acetone-*d6* at 25℃.


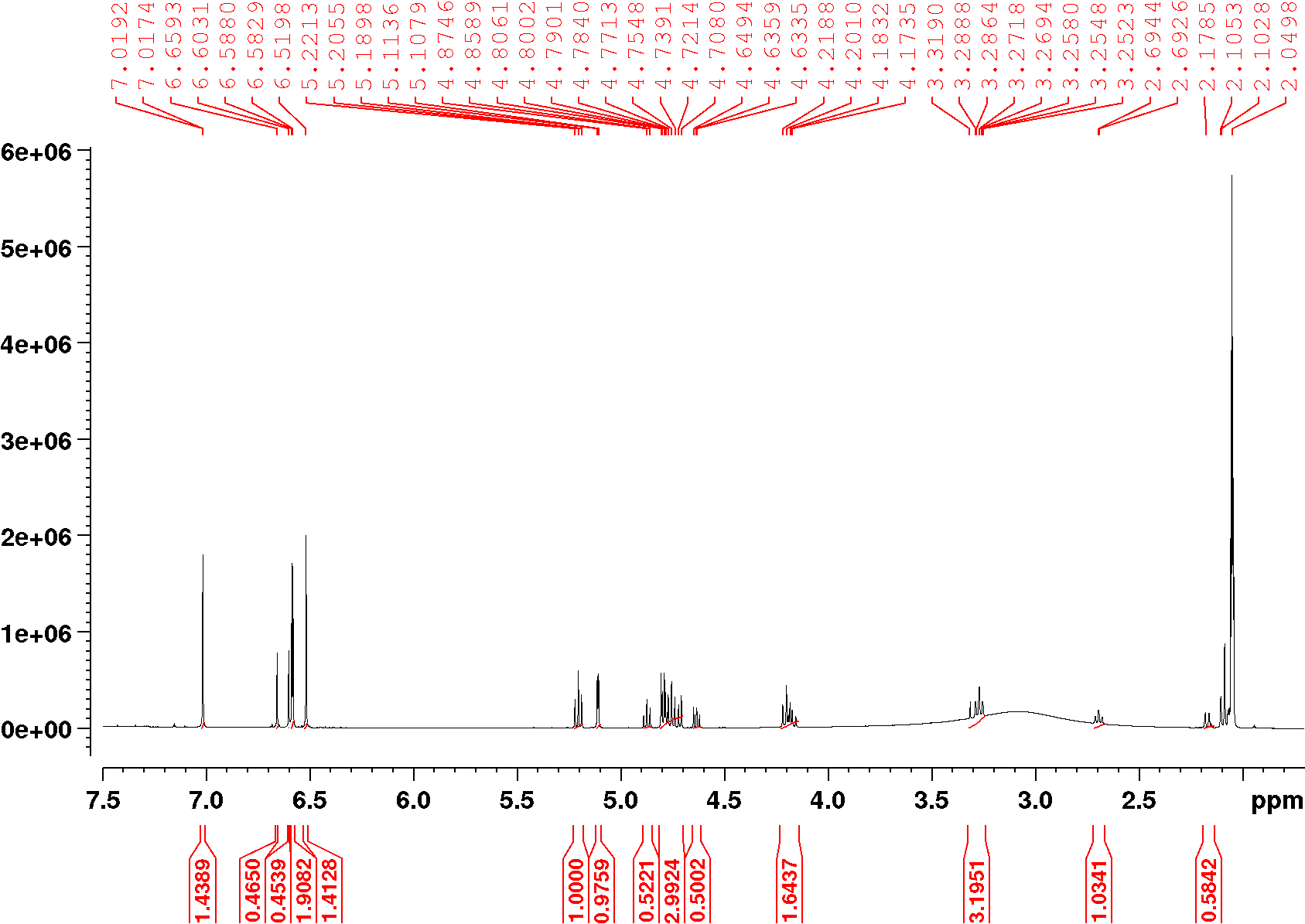


**Figure S11.** ^1^H NMR spectrum of punicalagin **9** measured in acetone-*d6* at 25℃.


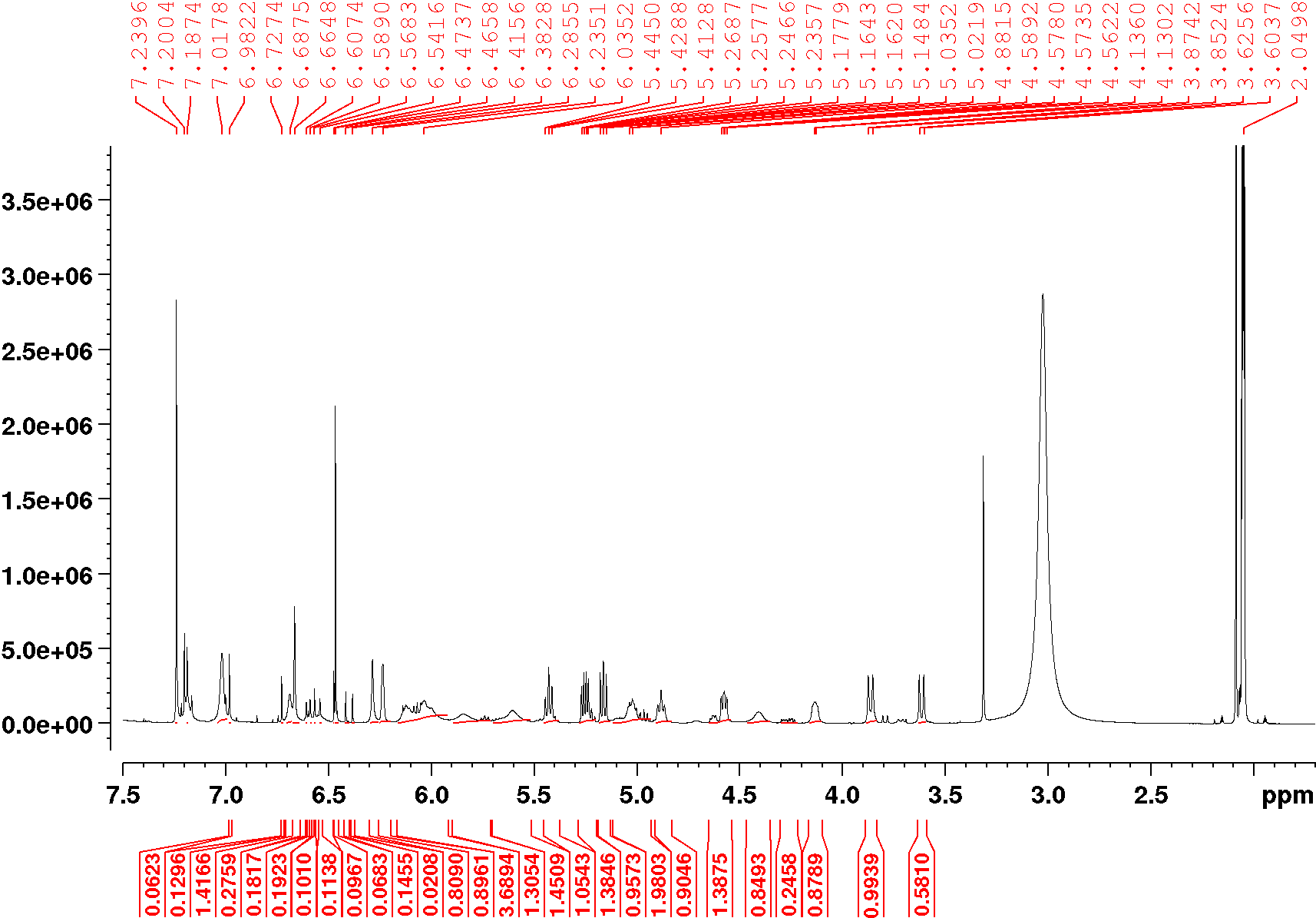


**Figure S12.** ^1^H NMR spectrum of oenothein B **10** measured in acetone-*d6* at 25℃.


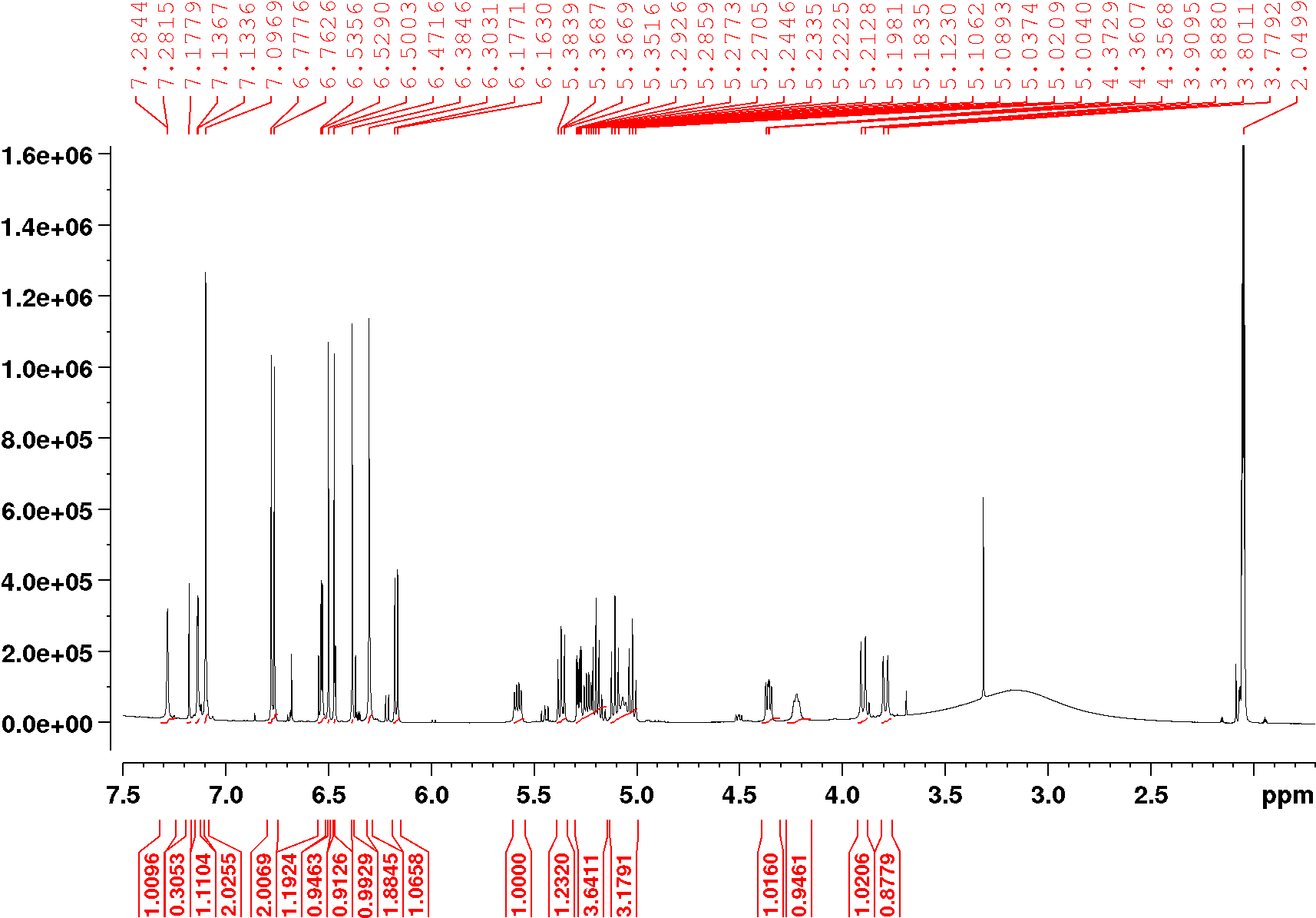


**Figure S13.** ^1^H NMR spectrum of sanguiin H-6 **11** measured in acetone-*d6* at 25℃.


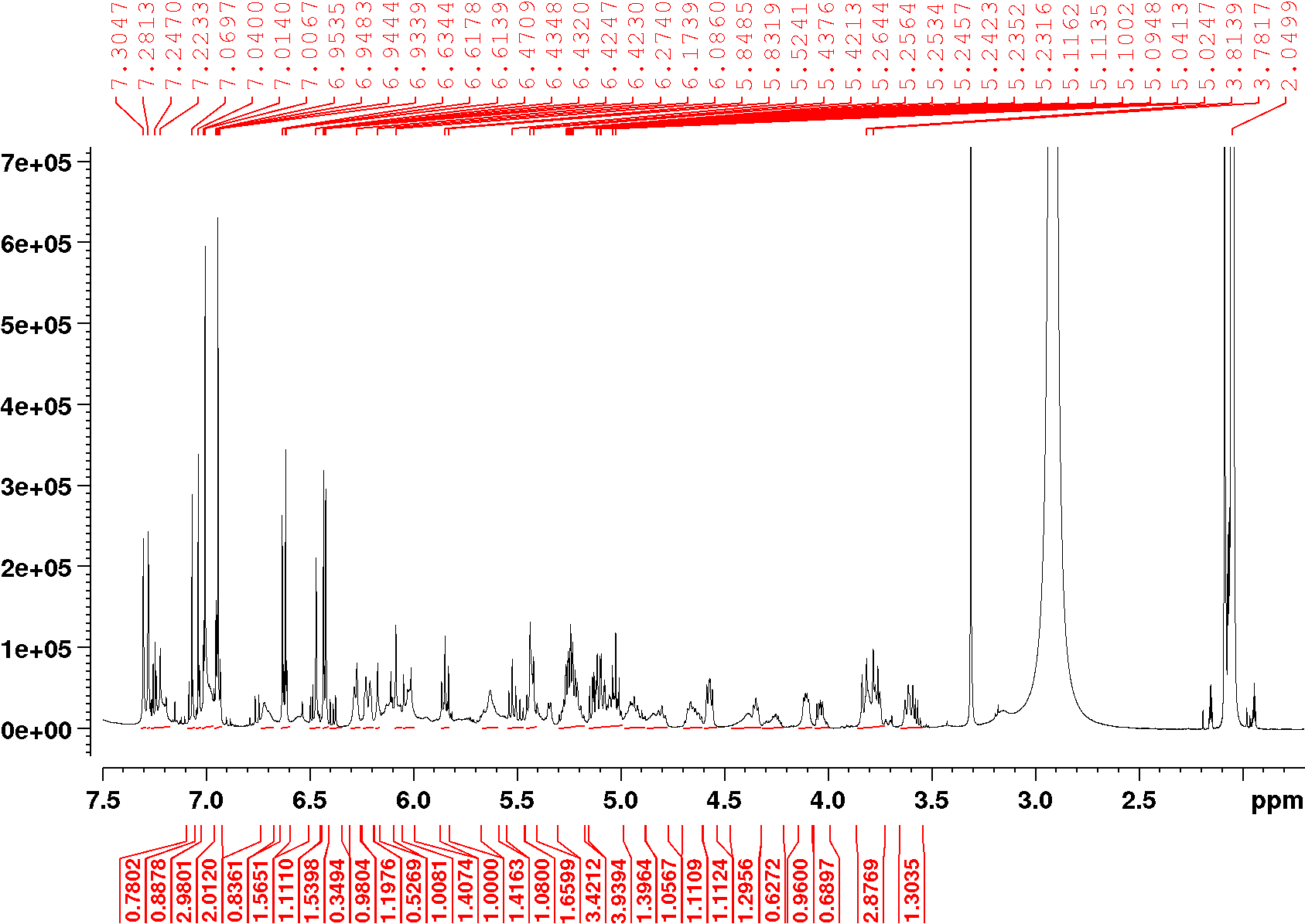


**Figure S14.** ^1^H NMR spectrum of oenothein A **12** measured in acetone-*d6* at 25℃.


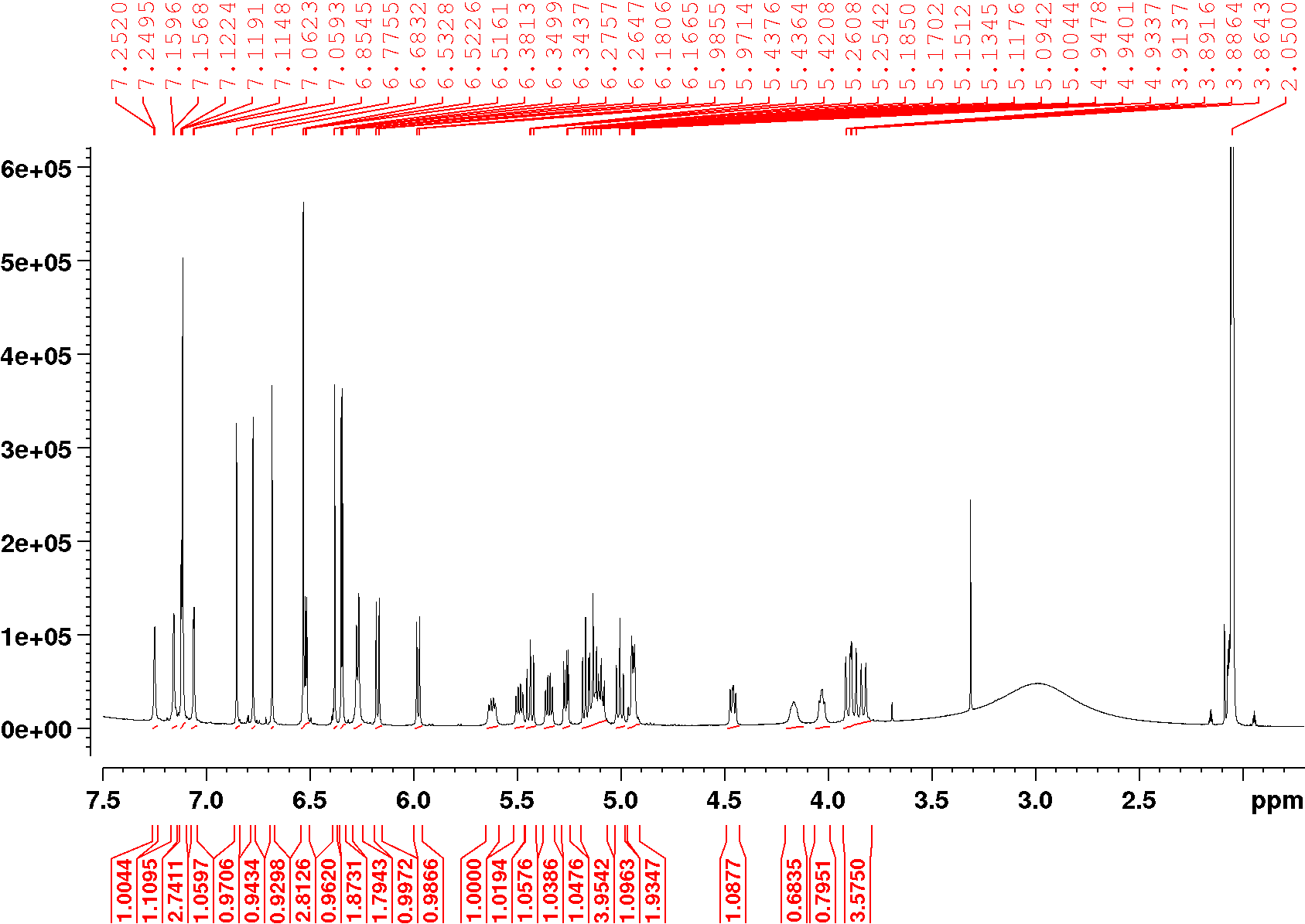


**Figure S15.** ^1^H NMR spectrum of lambertianin C **13** measured in acetone-*d6* at 25℃.
